# 2-Meth­oxy-9-phenoxy­acridine

**DOI:** 10.1107/S1600536810008962

**Published:** 2010-03-13

**Authors:** Damian Trzybiński, Beata Zadykowicz, Karol Krzymiński, Artur Sikorski, Jerzy Błażejowski

**Affiliations:** a Faculty of Chemistry, University of Gdańsk, J. Sobieskiego 18, 80-952 Gdańsk, Poland

## Abstract

The mol­ecules in the crystal structure of the title compound, C_20_H_15_NO_2_, form inversion dimers connected through the C—H⋯N and π–π inter­actions. These dimers are further linked by C—H⋯π inter­actions. The meth­oxy group is nearly coplanar with the acridine ring system [dihedral angle = 4.5 (1)°], whereas the phen­oxy fragment is nearly perpendicular to it [dihedral angle = 85.0 (1)°]. The mean planes of the acridine ring systems are either parallel or inclined at angles of 14.3 (1), 65.4 (1) and 67.3 (1)° in the crystal.

## Related literature

For general background to 9-phenoxy­acridines, see: Acheson (1973 ▶); Albert (1966 ▶); Chen *et al.* (2002 ▶); Demeunynck *et al.* (2001 ▶); Lebekhov & Samarin (1969 ▶); Ueyama *et al.* (2002 ▶). For related structures, see: Ebead *et al.* (2005 ▶); Sikorski *et al.* (2007 ▶). For inter­molecular inter­actions, see: Hunter *et al.* (2001 ▶); Mazik *et al.* (2000 ▶); Takahashi *et al.* (2001 ▶). For the synthesis, see: Acheson (1973 ▶); Chen *et al.* (2002 ▶); Duprè & Robinson (1945 ▶).
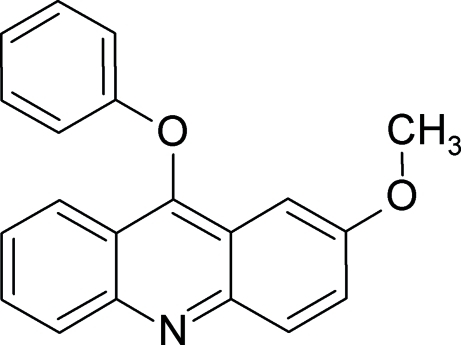


## Experimental

### 

#### Crystal data

C_20_H_15_NO_2_
*M* *_r_* = 301.33
Orthorhombic, 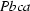

*a* = 8.3042 (2) Å
*b* = 15.5101 (4) Å
*c* = 24.0192 (6) Å
*V* = 3093.65 (13) Å^3^
*Z* = 8
Mo *K*α radiation
μ = 0.08 mm^−1^
*T* = 295 K
0.50 × 0.25 × 0.10 mm

#### Data collection

Oxford Diffraction Gemini R Ultra Ruby CCD diffractometer
Absorption correction: multi-scan (*CrysAlis RED*; Oxford Diffraction, 2008 ▶) *T*_min_ = 0.890, *T*_max_ = 0.994
56825 measured reflections
2747 independent reflections
2322 reflections with *I* > 2σ(*I*)
*R*_int_ = 0.024

#### Refinement

*R*[*F* ^2^ > 2σ(*F* ^2^)] = 0.029
*wR*(*F* ^2^) = 0.086
*S* = 1.10
2747 reflections
210 parameters
H-atom parameters constrained
Δρ_max_ = 0.15 e Å^−3^
Δρ_min_ = −0.11 e Å^−3^

### 

Data collection: *CrysAlis CCD* (Oxford Diffraction, 2008 ▶); cell refinement: *CrysAlis RED* (Oxford Diffraction, 2008 ▶); data reduction: *CrysAlis RED*; program(s) used to solve structure: *SHELXS97* (Sheldrick, 2008 ▶); program(s) used to refine structure: *SHELXL97* (Sheldrick, 2008 ▶); molecular graphics: *ORTEP-3* (Farrugia, 1997 ▶); software used to prepare material for publication: *SHELXL97* and *PLATON* (Spek, 2009 ▶).

## Supplementary Material

Crystal structure: contains datablocks global, I. DOI: 10.1107/S1600536810008962/ng2741sup1.cif

Structure factors: contains datablocks I. DOI: 10.1107/S1600536810008962/ng2741Isup2.hkl

Additional supplementary materials:  crystallographic information; 3D view; checkCIF report

## Figures and Tables

**Table 1:** Hydrogen-bond geometry (Å, °) *Cg*2 and *Cg*4 are the centroids of the C1–C4/C11/C12 and C18–C23 rings, respectively.

*D*—H⋯*A*	*D*—H	H⋯*A*	*D*⋯*A*	*D*—H⋯*A*
C19—H19⋯N10^i^	0.93	2.60	3.487 (2)	160
C6—H6⋯*Cg*4^ii^	0.93	2.80	3.459 (2)	129
C16—H16*B*⋯*Cg*4^iii^	0.96	2.94	3.658 (2)	133
C20—H20⋯*Cg*2^iv^	0.93	2.71	3.576 (2)	156

Symmetry codes: (i) 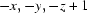; (ii) 
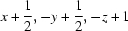
; (iii) 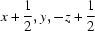; (iv) 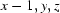.

**Table 2:** π–π inter­actions (Å,°) *Cg*1, *Cg*2 and *Cg*3 are the centroids of the C9/N10/C11–C14, C1–C4/C11/C12 and C5–C8/C13/C14 rings, respectively. *CgI*⋯*CgJ* is the distance between ring centroids. The dihedral angle is that between the planes of the rings *I* and *J. CgI*_Perp is the perpendicular distance of *CgI* from ring *J. CgI*_Offset is the distance between *CgI* and the perpendicular projection of *CgJ* on ring *I*.

*I*	*J*	*CgI*⋯*CgJ*	Dihedral angle	*CgI*_Perp	*CgI*_Offset
1	1^i^	3.984 (1)	0.0	3.569 (1)	1.770 (1)
2	3^i^	3.932 (1)	1.6	3.564 (1)	1.661 (1)
3	2^i^	3.932 (1)	1.6	3.541 (1)	1.707 (1)

Symmetry code: (i) –*x*, –*y*, –*z* + 1.

**Fig. 1.**
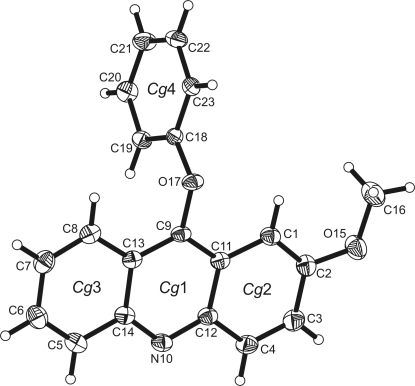
The molecular structure of the title compound showing the atom labeling scheme. Displacement ellipsoids are drawn at the 25% probability level and H atoms are shown as small spheres of arbitrary radius. Cg1, Cg2, Cg3 and Cg4 denote the ring centroids.

**Fig. 2.**
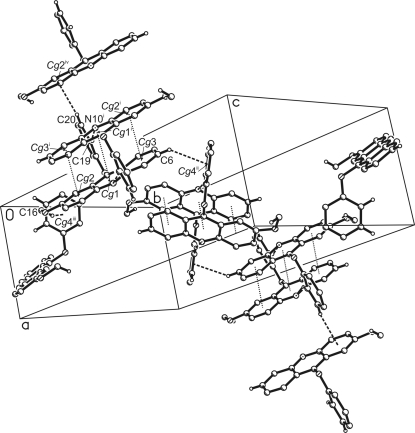
The arrangement of the molecules in the crystal structure. The C–H···N and C–H···π interactions are represented by dashed lines and π–π interactions by dotted lines. [Symmetry codes: (i) –x, –y, –z+1; (ii) x+1/2, –y+1/2, –z+1; (iii) x+1/2, y, –z+1/2; (iv) x–1, y, z.]

## supplementary crystallographic information

### Comment

9-Phenoxyacridines are convenient precursors of 9-substituted acridines owing to their excellent stability during storage at room temperature (Albert, 1966; Acheson, 1973); they effectively react with hydrochlorides of various amines to yield the respective 9-acridinamines. The compounds belonging to this group were proposed as fluorescent labels in medicinal diagnostics (Ueyama *et al.*, 2002) and checked for anti-bacterial (Lebekhov & Samarin, 1969) and anti-inflammatory (Chen *et al.*, 2002) activities. Here we demonstrate the structure of 9-phenoxyacridine substituted with the methoxy group at the acridine moiety; we investigated the parent molecule (i.e. 9-phenoxyacridine) earlier (Ebead *et al.*, 2005). Such substitution may affect spectral features of 9-phenoxyacridine and facilitate its conversion to medically interesting derivatives (Demeunynck *et al.*, 2001).

In the crystal structure, the inversely oriented molecules form dimers through π–π interactions involving acridine skeletons (Table 2, Fig. 2) and C(aromatic)–H···N interactions (Table 1, Fig. 2). These dimers are linked in the crystal lattice by C(aliphatic, aromatic)–H···π interactions (Table 1, Fig. 2). The C–H···N interactions are of the hydrogen bond type (Steiner, 1999). The C–H···π interactions (Takahashi *et al.*, 2001), like the π–π interactions (Hunter *et al.*, 2001) should be of an attractive nature. The crystal structure is stabilized by a network of these short-range specific interactions and by non-specific dispersive interactions between adjacent molecules.

In the title compound (Fig. 1), the bond lengths and angles characterizing the geometry of the acridine moiety are typical of acridine based derivatives (Ebead *et al.*, 2005; Sikorski *et al.*, 2007). With a respective average deviation from planarity of 0.0147 (2) Å and 0.0072 (2) Å, the acridine and benzene ring systems are oriented at 85.0 (1)°, i.e. they are nearly perpendicular to each other. On the other hand, the methoxy group is almost co-planar with the acridine skeleton (the angle between the mean plane of the acridine moiety and the plane delineated by C2, O15 and C16 is 4.5 (1)°). C9, N10 and O17 are arranged almost linearly (N10···C9–O17 angle = 174.9 (1)°). The mean planes of the adjacent acridine moieties are either parallel (they remain at an angle of 0.0 (1)° – in dimers) or inclined at angles of 14.3 (1)°, 65.4 (1)° and 67.3 (1)° in the lattice. The molecular structure of the compound investigated is similar to that of 9-phenoxyacridine (Ebead *et al.*, 2005).

### Experimental

2-Methoxy-9-chloroacridine was prepared by heating 2-[(2-methoxyphenyl)amino]benzoic acid, obtained as described elsewhere (Acheson, 1973), with a sevenfold molar excess of POCl_3_ (400 K, 3 h). The excess POCl_3_ was subsequently removed under reduced pressure. The residue was dispersed in CHCl_3_, stirred in the presence of a mixture of ice and aqueous ammonia, separated by filtration and dried. The crude product was purified chromatographically (neutral Al_2_O_3_, CHCl_3_/toluene, 1/1 v/v). The obtained 2-methoxy-9-chloroacridine was added to the solution of NaOH in phenol (sevenfold molar excess) in equimolar to NaOH amount, at 373 K under continuous stirring. The reactant mixture was kept at 373 K for 1.5 h, subsequently poured into 2M aq NaOH and stored at room temperature overnight. The precipitate was separated by filtration, washed with water and dried (Duprè & Robinson, 1945; Chen *et al.*, 2002). Light-brown crystals of 2-methoxy-9-phenoxyacridine suitable for X-Ray investigations were grown from absolute ethanol solution (m.p. 415-417 K).

### Refinement

H atoms were positioned geometrically, with C—H = 0.93 Å (aromatic) or 0.96 Å (methyl), and constrained to ride on their parent atoms with *U*_iso_(H) = 1.2*U*_eq_(C) (aromatic) or *U*_iso_(H) = 1.5*U*_eq_(C) (methyl).

### Crystal data

**Table:**

C_20_H_15_NO_2_	*F*(000) = 1264
*M**_r_* = 301.33	*D*_x_ = 1.294 Mg m^−^^3^
Orthorhombic, *P**b**c**a*	Mo *K*α radiation, λ = 0.71073 Å
Hall symbol: -P 2ac 2ab	Cell parameters from 32561 reflections
*a* = 8.3042 (2) Å	θ = 3.0–29.3°
*b* = 15.5101 (4) Å	µ = 0.08 mm^−^^1^
*c* = 24.0192 (6) Å	*T* = 295 K
*V* = 3093.65 (13) Å^3^	Plate, light-brown
*Z* = 8	0.50 × 0.25 × 0.10 mm

### Data collection

**Table:**

Oxford Diffraction Gemini R Ultra Ruby CCD diffractometer	2747 independent reflections
Radiation source: Enhance (Mo) X-ray Source	2322 reflections with *I* > 2σ(*I*)
graphite	*R*_int_ = 0.024
Detector resolution: 10.4002 pixels mm^-1^	θ_max_ = 25.1°, θ_min_ = 3.0°
ω scans	*h* = −9→9
Absorption correction: multi-scan (*CrysAlis RED*; Oxford Diffraction, 2008)	*k* = −18→18
*T*_min_ = 0.890, *T*_max_ = 0.994	*l* = −28→28
56825 measured reflections	

### Refinement

**Table:**

Refinement on *F*^2^	Secondary atom site location: difference Fourier map
Least-squares matrix: full	Hydrogen site location: inferred from neighbouring sites
*R*[*F*^2^ > 2σ(*F*^2^)] = 0.029	H-atom parameters constrained
*wR*(*F*^2^) = 0.086	*w* = 1/[σ^2^(*F*_o_^2^) + (0.048*P*)^2^ + 0.2828*P*] where *P* = (*F*_o_^2^ + 2*F*_c_^2^)/3
*S* = 1.10	(Δ/σ)_max_ < 0.001
2747 reflections	Δρ_max_ = 0.15 e Å^−^^3^
210 parameters	Δρ_min_ = −0.11 e Å^−^^3^
0 restraints	Extinction correction: *SHELXL97* (Sheldrick, 2008), Fc^*^=kFc[1+0.001xFc^2^λ^3^/sin(2θ)]^-1/4^
Primary atom site location: structure-invariant direct methods	Extinction coefficient: 0.0046 (6)

### Special details

**Table:**

Geometry. All esds (except the esd in the dihedral angle between two l.s. planes) are estimated using the full covariance matrix. The cell esds are taken into account individually in the estimation of esds in distances, angles and torsion angles; correlations between esds in cell parameters are only used when they are defined by crystal symmetry. An approximate (isotropic) treatment of cell esds is used for estimating esds involving l.s. planes.

### Fractional atomic coordinates and isotropic or equivalent isotropic displacement parameters (Å^2^)

**Table:**

	*x*	*y*	*z*	*U*_iso_*/*U*_eq_	
C1	0.18244 (13)	0.00760 (7)	0.34444 (4)	0.0442 (3)	
H1	0.1362	0.0364	0.3145	0.053*	
C2	0.26680 (14)	−0.06681 (7)	0.33602 (5)	0.0498 (3)	
C3	0.33776 (16)	−0.11161 (8)	0.38157 (5)	0.0560 (3)	
H3	0.3938	−0.1626	0.3751	0.067*	
C4	0.32483 (15)	−0.08118 (8)	0.43386 (5)	0.0525 (3)	
H4	0.3731	−0.1112	0.4629	0.063*	
C5	0.13635 (15)	0.12529 (8)	0.56536 (5)	0.0522 (3)	
H5	0.1883	0.0946	0.5934	0.063*	
C6	0.05228 (16)	0.19741 (9)	0.57843 (5)	0.0591 (3)	
H6	0.0471	0.2155	0.6153	0.071*	
C7	−0.02759 (15)	0.24540 (8)	0.53666 (5)	0.0573 (3)	
H7	−0.0854	0.2945	0.5464	0.069*	
C8	−0.02090 (13)	0.22067 (7)	0.48256 (5)	0.0478 (3)	
H8	−0.0737	0.2530	0.4555	0.057*	
C9	0.08142 (12)	0.11619 (6)	0.41230 (4)	0.0383 (2)	
N10	0.23001 (11)	0.02343 (6)	0.49857 (4)	0.0460 (2)	
C11	0.16565 (11)	0.04083 (6)	0.39947 (4)	0.0389 (2)	
C12	0.23839 (12)	−0.00369 (7)	0.44563 (4)	0.0416 (3)	
C13	0.06662 (12)	0.14545 (7)	0.46691 (4)	0.0397 (3)	
C14	0.14617 (12)	0.09591 (7)	0.50938 (4)	0.0421 (3)	
O15	0.29423 (13)	−0.10503 (6)	0.28557 (3)	0.0676 (3)	
C16	0.2364 (2)	−0.06180 (10)	0.23706 (5)	0.0749 (4)	
H16A	0.2695	−0.0929	0.2045	0.112*	
H16B	0.2799	−0.0045	0.2358	0.112*	
H16C	0.1210	−0.0589	0.2382	0.112*	
O17	0.02052 (8)	0.16615 (5)	0.36901 (3)	0.0441 (2)	
C18	−0.13908 (11)	0.15439 (6)	0.35297 (4)	0.0352 (2)	
C19	−0.24472 (13)	0.10027 (6)	0.38029 (4)	0.0410 (3)	
H19	−0.2123	0.0697	0.4117	0.049*	
C20	−0.40022 (14)	0.09241 (8)	0.35996 (5)	0.0508 (3)	
H20	−0.4727	0.0561	0.3779	0.061*	
C21	−0.44883 (14)	0.13772 (9)	0.31347 (5)	0.0572 (3)	
H21	−0.5530	0.1314	0.2998	0.069*	
C22	−0.34193 (15)	0.19255 (9)	0.28733 (5)	0.0552 (3)	
H22	−0.3745	0.2235	0.2561	0.066*	
C23	−0.18710 (13)	0.20176 (7)	0.30720 (4)	0.0441 (3)	
H23	−0.1158	0.2395	0.2900	0.053*	

### Atomic displacement parameters (Å^2^)

**Table:**

	*U*^11^	*U*^22^	*U*^33^	*U*^12^	*U*^13^	*U*^23^
C1	0.0460 (6)	0.0434 (6)	0.0434 (6)	−0.0019 (5)	−0.0025 (5)	0.0079 (5)
C2	0.0564 (7)	0.0440 (6)	0.0489 (7)	−0.0009 (5)	0.0051 (5)	0.0015 (5)
C3	0.0648 (8)	0.0420 (6)	0.0612 (8)	0.0114 (5)	0.0039 (6)	0.0071 (5)
C4	0.0579 (7)	0.0453 (6)	0.0544 (7)	0.0097 (5)	−0.0032 (5)	0.0141 (5)
C5	0.0596 (7)	0.0521 (7)	0.0450 (6)	−0.0066 (6)	−0.0065 (5)	0.0042 (5)
C6	0.0686 (8)	0.0579 (8)	0.0508 (7)	−0.0073 (6)	0.0007 (6)	−0.0078 (6)
C7	0.0558 (7)	0.0500 (7)	0.0661 (8)	0.0012 (6)	0.0028 (6)	−0.0079 (6)
C8	0.0427 (6)	0.0436 (6)	0.0570 (7)	−0.0004 (5)	−0.0019 (5)	0.0039 (5)
C9	0.0339 (5)	0.0377 (5)	0.0432 (6)	−0.0040 (4)	−0.0049 (4)	0.0119 (4)
N10	0.0487 (5)	0.0440 (5)	0.0452 (5)	0.0003 (4)	−0.0051 (4)	0.0099 (4)
C11	0.0354 (5)	0.0374 (5)	0.0438 (6)	−0.0039 (4)	−0.0014 (4)	0.0091 (4)
C12	0.0405 (5)	0.0401 (6)	0.0442 (6)	−0.0017 (4)	−0.0021 (4)	0.0095 (5)
C13	0.0343 (5)	0.0380 (6)	0.0469 (6)	−0.0058 (4)	−0.0019 (4)	0.0067 (4)
C14	0.0414 (6)	0.0407 (6)	0.0443 (6)	−0.0071 (4)	−0.0030 (4)	0.0068 (5)
O15	0.0937 (7)	0.0575 (5)	0.0515 (5)	0.0130 (5)	0.0051 (5)	−0.0031 (4)
C16	0.1016 (11)	0.0740 (9)	0.0492 (8)	0.0065 (8)	−0.0016 (7)	−0.0043 (7)
O17	0.0378 (4)	0.0443 (4)	0.0502 (4)	−0.0016 (3)	−0.0052 (3)	0.0173 (3)
C18	0.0366 (5)	0.0341 (5)	0.0349 (5)	0.0029 (4)	−0.0004 (4)	−0.0010 (4)
C19	0.0472 (6)	0.0367 (5)	0.0393 (5)	−0.0018 (4)	−0.0032 (4)	0.0043 (4)
C20	0.0470 (6)	0.0497 (7)	0.0556 (7)	−0.0106 (5)	−0.0013 (5)	0.0020 (5)
C21	0.0472 (7)	0.0685 (8)	0.0560 (7)	−0.0050 (6)	−0.0155 (5)	0.0019 (6)
C22	0.0540 (7)	0.0698 (8)	0.0418 (6)	0.0042 (6)	−0.0099 (5)	0.0111 (6)
C23	0.0452 (6)	0.0500 (6)	0.0369 (5)	0.0033 (5)	0.0037 (4)	0.0086 (5)

### Geometric parameters (Å, °)

**Table:**

C1—C2	1.3651 (16)	N10—C12	1.3413 (14)
C1—C11	1.4256 (15)	N10—C14	1.3476 (14)
C1—H1	0.9300	C11—C12	1.4391 (14)
C2—O15	1.3681 (14)	C13—C14	1.4378 (14)
C2—C3	1.4237 (17)	O15—C16	1.4276 (16)
C3—C4	1.3460 (17)	C16—H16A	0.9600
C3—H3	0.9300	C16—H16B	0.9600
C4—C12	1.4282 (16)	C16—H16C	0.9600
C4—H4	0.9300	O17—C18	1.3922 (12)
C5—C6	1.3554 (19)	C18—C19	1.3801 (14)
C5—C14	1.4220 (16)	C18—C23	1.3812 (14)
C5—H5	0.9300	C19—C20	1.3859 (15)
C6—C7	1.4144 (18)	C19—H19	0.9300
C6—H6	0.9300	C20—C21	1.3797 (17)
C7—C8	1.3560 (16)	C20—H20	0.9300
C7—H7	0.9300	C21—C22	1.3805 (18)
C8—C13	1.4250 (16)	C21—H21	0.9300
C8—H8	0.9300	C22—C23	1.3788 (16)
C9—O17	1.3920 (12)	C22—H22	0.9300
C9—C13	1.3934 (14)	C23—H23	0.9300
C9—C11	1.3965 (15)		
			
C2—C1—C11	119.54 (10)	C4—C12—C11	117.54 (10)
C2—C1—H1	120.2	C9—C13—C8	124.06 (9)
C11—C1—H1	120.2	C9—C13—C14	116.95 (9)
C1—C2—O15	125.66 (10)	C8—C13—C14	118.99 (10)
C1—C2—C3	120.75 (11)	N10—C14—C5	118.62 (10)
O15—C2—C3	113.59 (10)	N10—C14—C13	123.13 (10)
C4—C3—C2	120.85 (11)	C5—C14—C13	118.25 (10)
C4—C3—H3	119.6	C2—O15—C16	117.61 (10)
C2—C3—H3	119.6	O15—C16—H16A	109.5
C3—C4—C12	121.32 (10)	O15—C16—H16B	109.5
C3—C4—H4	119.3	H16A—C16—H16B	109.5
C12—C4—H4	119.3	O15—C16—H16C	109.5
C6—C5—C14	120.86 (11)	H16A—C16—H16C	109.5
C6—C5—H5	119.6	H16B—C16—H16C	109.5
C14—C5—H5	119.6	C9—O17—C18	118.66 (7)
C5—C6—C7	120.77 (11)	C19—C18—C23	121.24 (9)
C5—C6—H6	119.6	C19—C18—O17	123.59 (9)
C7—C6—H6	119.6	C23—C18—O17	115.17 (9)
C8—C7—C6	120.77 (12)	C18—C19—C20	118.57 (10)
C8—C7—H7	119.6	C18—C19—H19	120.7
C6—C7—H7	119.6	C20—C19—H19	120.7
C7—C8—C13	120.35 (11)	C21—C20—C19	120.86 (11)
C7—C8—H8	119.8	C21—C20—H20	119.6
C13—C8—H8	119.8	C19—C20—H20	119.6
O17—C9—C13	119.33 (9)	C20—C21—C22	119.58 (11)
O17—C9—C11	118.88 (9)	C20—C21—H21	120.2
C13—C9—C11	121.64 (9)	C22—C21—H21	120.2
C12—N10—C14	118.10 (9)	C23—C22—C21	120.40 (10)
C9—C11—C1	123.79 (9)	C23—C22—H22	119.8
C9—C11—C12	116.22 (9)	C21—C22—H22	119.8
C1—C11—C12	119.99 (9)	C22—C23—C18	119.31 (10)
N10—C12—C4	118.53 (9)	C22—C23—H23	120.3
N10—C12—C11	123.94 (10)	C18—C23—H23	120.3
			
C11—C1—C2—O15	−178.96 (10)	C11—C9—C13—C14	1.82 (14)
C11—C1—C2—C3	0.15 (17)	C7—C8—C13—C9	−179.10 (10)
C1—C2—C3—C4	−0.73 (19)	C7—C8—C13—C14	0.59 (16)
O15—C2—C3—C4	178.49 (11)	C12—N10—C14—C5	179.97 (9)
C2—C3—C4—C12	0.68 (19)	C12—N10—C14—C13	−0.39 (15)
C14—C5—C6—C7	−0.13 (19)	C6—C5—C14—N10	−179.41 (11)
C5—C6—C7—C8	−0.47 (19)	C6—C5—C14—C13	0.93 (17)
C6—C7—C8—C13	0.22 (18)	C9—C13—C14—N10	−1.08 (14)
O17—C9—C11—C1	−5.34 (14)	C8—C13—C14—N10	179.21 (9)
C13—C9—C11—C1	179.14 (9)	C9—C13—C14—C5	178.57 (9)
O17—C9—C11—C12	174.41 (8)	C8—C13—C14—C5	−1.14 (14)
C13—C9—C11—C12	−1.11 (14)	C1—C2—O15—C16	3.06 (18)
C2—C1—C11—C9	−179.81 (10)	C3—C2—O15—C16	−176.11 (12)
C2—C1—C11—C12	0.45 (15)	C13—C9—O17—C18	−88.82 (11)
C14—N10—C12—C4	−179.03 (10)	C11—C9—O17—C18	95.56 (11)
C14—N10—C12—C11	1.18 (15)	C9—O17—C18—C19	4.98 (14)
C3—C4—C12—N10	−179.88 (11)	C9—O17—C18—C23	−175.51 (9)
C3—C4—C12—C11	−0.07 (17)	C23—C18—C19—C20	1.71 (16)
C9—C11—C12—N10	−0.46 (15)	O17—C18—C19—C20	−178.82 (9)
C1—C11—C12—N10	179.31 (10)	C18—C19—C20—C21	−0.10 (17)
C9—C11—C12—C4	179.75 (9)	C19—C20—C21—C22	−0.95 (19)
C1—C11—C12—C4	−0.49 (15)	C20—C21—C22—C23	0.4 (2)
O17—C9—C13—C8	6.01 (15)	C21—C22—C23—C18	1.14 (18)
C11—C9—C13—C8	−178.49 (9)	C19—C18—C23—C22	−2.23 (16)
O17—C9—C13—C14	−173.68 (8)	O17—C18—C23—C22	178.25 (10)

### Hydrogen-bond geometry (Å, °)

**Table:**

Cg2 and Cg4 are the centroids of the C1–C4/C11/C12 and C18–C23 rings, respectively.

**Table:**

*D*—H···*A*	*D*—H	H···*A*	*D*···*A*	*D*—H···*A*
C19—H19···N10^i^	0.93	2.60	3.487 (2)	160
C6—H6···*Cg*4^ii^	0.93	2.80	3.459 (2)	129
C16—H16B···*Cg*4^iii^	0.96	2.94	3.658 (2)	133
C20—H20···*Cg*2^iv^	0.93	2.71	3.576 (2)	156

Symmetry codes:  (i) −*x*, −*y*, −*z*+1; (ii) *x*+1/2, −*y*+1/2, −*z*+1; (iii) *x*+1/2, *y*, −*z*+1/2; (iv) *x*−1, *y*, *z*.

### Table 2 π–π interactions (Å,°)

*Cg*1, *Cg*2 and *Cg*3 are the centroids of the C9/N10/C11–C14, C1–C4/C11/C12 and C5–C8/C13/C14 rings, respectively. *CgI*···*CgJ* is the distance between ring centroids. The dihedral angle is that between the planes of the rings *I* and *J*. *CgI*_Perp is the perpendicular distance of *CgI* from ring *J*. *CgI*_Offset is the distance between *CgI* and the perpendicular projection of *CgJ* on ring *I*.

**Table:**

*I*	*J*	*CgI*···*CgJ*	Dihedral angle	*CgI*_Perp	*CgI*_Offset
1	1^i^	3.984 (1)	0.0	3.569 (1)	1.770 (1)
2	3^i^	3.932 (1)	1.6	3.564 (1)	1.661 (1)
3	2^i^	3.932 (1)	1.6	3.541 (1)	1.707 (1)

Symmetry codes: (i) –*x*, –*y*, –*z*+1.

## References

[bb1] Acheson, R. M. (1973). In *Acridines*, 2nd ed. New York: Interscience.

[bb2] Albert, A. (1966). In *The Acridines*, London: Edward Arnold.

[bb3] Chen, Y.-L., Lu, C.-M., Chen, I.-L., Tsao, L.-T. & Wang, J.-P. (2002). *J. Med. Chem.* **45**, 4689–4694. 10.1021/jm020102v 12361395

[bb4] Demeunynck, M., Charmantray, F. & Martelli, A. (2001). *Curr. Pharm. Des.* **7**, 1703–1724. 10.2174/1381612013397131 11562307

[bb5] Duprè, D. J. & Robinson, F. A. (1945). *J. Chem. Soc.* pp. 549–551.

[bb6] Ebead, Y., Sikorski, A., Krzymiński, K., Lis, T. & Błażejowski, J. (2005). *Acta Cryst.* C**61**, o85–o87. 10.1107/S0108270104025156 15695918

[bb7] Farrugia, L. J. (1997). *J. Appl. Cryst.* **30**, 565.

[bb8] Hunter, C. A., Lawson, K. R., Perkins, J. & Urch, C. J. (2001). *J. Chem. Soc. Perkin Trans. 2*, pp. 651–669.

[bb9] Lebekhov, A. C. & Samarin, A. S. (1969). *Khim. Geterotsikl. Soedin.* **5**, 838–841.

[bb10] Mazik, M., Bläser, D. & Boese, R. (2000). *Tetrahedron Lett.* **41**, 5827–5831.

[bb11] Oxford Diffraction (2008). *CrysAlis CCD* and *CrysAlis RED* Oxford Diffraction Ltd, Yarnton, England.

[bb12] Sheldrick, G. M. (2008). *Acta Cryst.* **A**64, 112–122. 10.1107/S0108767307043930 18156677

[bb13] Sikorski, A., Kowalska, K., Krzymiński, K. & Błażejowski, J. (2007). *Acta Cryst.* E**63**, o2670–o2672.

[bb14] Spek, A. L. (2009). *Acta Cryst.* D**65**, 148–155. 10.1107/S090744490804362X PMC2631630 19171970

[bb15] Takahashi, O., Kohno, Y., Iwasaki, S., Saito, K., Iwaoka, M., Tomada, S., Umezawa, Y., Tsuboyama, S. & Nishio, M. (2001). *Bull. Chem. Soc. Jpn*, **74**, 2421–2430.

[bb16] Ueyama, H., Takagi, M. & Takenaka, S. (2002). *Analyst*, **127**, 886–888. 10.1039/b204019k 12173644

